# Probing the Structures of Viral RNA Regulatory Elements with SHAPE and Related Methodologies

**DOI:** 10.3389/fmicb.2017.02634

**Published:** 2018-01-09

**Authors:** Jason W. Rausch, Joanna Sztuba-Solinska, Stuart F. J. Le Grice

**Affiliations:** ^1^Basic Research Laboratory, National Cancer Institute, Frederick, MD, United States; ^2^Department of Biological Sciences, Auburn University, Auburn, AL, United States

**Keywords:** SHAPE, RNA structure, secondary structure, chemical probing, viral RNA

## Abstract

Viral RNAs were selected by evolution to possess maximum functionality in a minimal sequence. Depending on the classification of the virus and the type of RNA in question, viral RNAs must alternately be replicated, spliced, transcribed, transported from the nucleus into the cytoplasm, translated and/or packaged into nascent virions, and in most cases, provide the sequence and structural determinants to facilitate these processes. One consequence of this compact multifunctionality is that viral RNA structures can be exquisitely complex, often involving intermolecular interactions with RNA or protein, intramolecular interactions between sequence segments separated by several thousands of nucleotides, or specialized motifs such as pseudoknots or kissing loops. The fluidity of viral RNA structure can also present a challenge when attempting to characterize it, as genomic RNAs especially are likely to sample numerous conformations at various stages of the virus life cycle. Here we review advances in chemoenzymatic structure probing that have made it possible to address such challenges with respect to *cis*-acting elements, full-length viral genomes and long non-coding RNAs that play a major role in regulating viral gene expression.

## Introduction

As a consequence of constant improvements in screening technology (Connelly et al., 2017), recent years have witnessed a resurgence in efforts to target RNA with small molecules, evidenced by the discovery of ligands capable of stimulating exon skipping (Luo and Disney, 2014) and others directed against a bacterial riboswitch (Howe et al., 2015), micro RNA (miRNA) linked to hepatocellular carcinoma (Childs-Disney and Disney, 2016) and RNA repeats associated with spinocerebellar ataxia type 10 (Yang et al., 2016). Viral RNA genomes and virus-coded RNAs provide a vast source of potential therapeutic targets in the form of *cis-*acting regulatory elements that mediate their transcription, nuclear transport, translation, replication and packaging. Effective therapeutic targeting of these elements, which include highly specialized structural motifs such as 5′-3′ interactions, pseudoknots, riboswitches and triple helices, would render the virus or viruses in question incapable of replication and completely non-pathogenic.

Implicit in these emerging opportunities is detailed knowledge of the structures of pertinent RNA regulatory elements, their interactions with other virus- and/or host-associated factors (both protein and nucleic acid) and variations in structures and binding partners associated with localization to different biological compartments. These are especially intriguing challenges for retroviral RNA genomes, which are synthesized in the nucleus, transported to the cytoplasm where they provide the template for translation, and are packaged into the budding virion.

Structural characterization of such complex RNAs requires highly robust and reproducible biochemical techniques, the current gold standard of which is selective 2′-hydroxyl acylation analyzed by primer extension, or SHAPE (Merino et al., 2005; Wilkinson et al., 2006). Unlike almost any other biochemical or biophysical methodology, SHAPE may be used to map the secondary structure of an RNA of virtually any length to single nucleotide resolution. In conjunction with capillary electrophoresis, this technique was used to elegantly map the secondary structure of the HIV RNA genome in its entirety (CE-SHAPE; Watts et al., 2009). The resulting structural model was later refined by SHAPE and mutational profiling (SHAPE-MaP), a more sensitive, higher throughput variant of the technique that exploits the power of next generation sequencing (Siegfried et al., 2014). SHAPE-MaP was also used to structurally characterize Kaposi's sarcoma-associated herpes virus (KSHV) polyadenylated nuclear long non-coding (lnc) RNA (PAN) and map regulatory protein binding sites (Sztuba-Solinska et al., 2017).

The capacity of a regulatory RNA to assume alternative configurations in the absence of protein factors presents a unique challenge in SHAPE-based structural studies, since a mixed population is being sampled. This problem can be solved by using native gel electrophoresis to fractionate RNA conformers, which can then be probed separately *in situ*, extracted, and subjected to subsequent processing as in conventional CE-SHAPE. This in-gel SHAPE technique has been used to structurally characterize monomeric and dimeric variants of retroviral RNA UTRs (Kenyon et al., 2013) as well as distinct conformers of the HIV-1 RRE (Sherpa et al., 2015). Temporal variation in RNA structure, such as that observed upon HIV-2 RRE RNA folding, can be more problematic. In one such instance, secondary structural models for individual conformers were mathematically extracted from conventional SHAPE measurements and using other experimental parameters to be described (Lusvarghi et al., 2013a). In the context of long RNAs, discrete structural motifs such as hairpins, kissing loops and pseudoknots can be verified and analyzed by antisense-interfered SHAPE (aiSHAPE), a technique in which short oligonucleotides are hybridized to part of a putative motif while effects of this hybridization on the remainder of the motif are characterized by SHAPE (Legiewicz et al., 2010). These technologies, together with structural features of select viral RNA elements they were used to characterize, are reviewed here.

## Structural characterization of RNA transport elements using SHAPE

Post-transcriptional control is necessary for expression of cellular and viral mRNAs, and is mediated by interactions of messenger ribonucleoproteins with transport receptors and components of the nuclear pore complex. Typically, RNA binding proteins in these complexes selectively recognize individual or collections of *cis*-acting motifs embedded in these mRNAs, the secondary and tertiary structures of which are as or more important than primary sequence in regulating nuclear export. Structural characterization of these elements has been greatly facilitated by the advent of SHAPE (Wilkinson et al., 2006). Unlike related methods, wherein probing reagents only cleave at or modify a subset of the four ribonucleotide bases, the two reagents primarily used in SHAPE (NMIA, 1M7) selectively acylate single stranded ribonucleotides at the 2′-hydroxyl position. Moreover, acylated ribonucleotides impede polymerase translocation during reverse transcription of modified RNAs, which serves to create a record of RNA acylation sites in derivative cDNA library populations.

In conventional SHAPE protocols, folded RNAs are modified by acylating reagent and then reverse transcribed from a 5′-^32^P or fluorescently labeled primer. The resulting cDNA library is fractionated by denaturing polyacrylamide gel or capillary electrophoresis (PAGE, CE, respectively), the bands/peaks are matched with corresponding ribonucleotides, and their intensities are measured. Band/peak intensity measurements are subsequently transformed into reactivity values that reflect the frequency with which the corresponding ribonucleotides have been acylated, and thus, the likelihood that they are single stranded in the context of the folded RNA. The collection of reactivity values is then inputted into RNAstructure software (Mathews, 2006), which converts the values into pseudo-energy constraints that are merged with empirically determined thermodynamic parameters and processed by an RNA folding algorithm to produce secondary structural models ranked by free energy. A more comprehensive description of the SHAPE methodology and data processing is provided elsewhere (Lusvarghi et al., 2013b), while the secondary structural models generated by applying SHAPE, CE-SHAPE, and/or related technologies to select RNA transport elements are described below.

### HIV-1 RRE: overall structure and the role of S-1

Singly spliced or unspliced HIV genomic RNA must be exported from the nucleus either for translation of the Gag-Pol polyprotein or packaging into nascent virions. This process is mediated by the specific interaction between Rev, a viral protein expressed in the early stages of infection, and the Rev response element (RRE), a highly structured RNA motif embedded within the envelope gene in HIV transcripts (Feinberg et al., 1986; Pavlakis and Felber, 1990). *In vitro* analysis indicates that six or more Rev molecules bind the RRE, together with two molecules of the cellular protein Crm1 in a cooperative and coordinated manner (Daugherty et al., 2010; Booth et al., 2014). The Rev_6_-RRE-Crm1 complex bypasses the RNA spicing machinery and is exported from the nucleus *via* the Crm1 export pathway (Askjaer et al., 1998).

**Figure 1:**
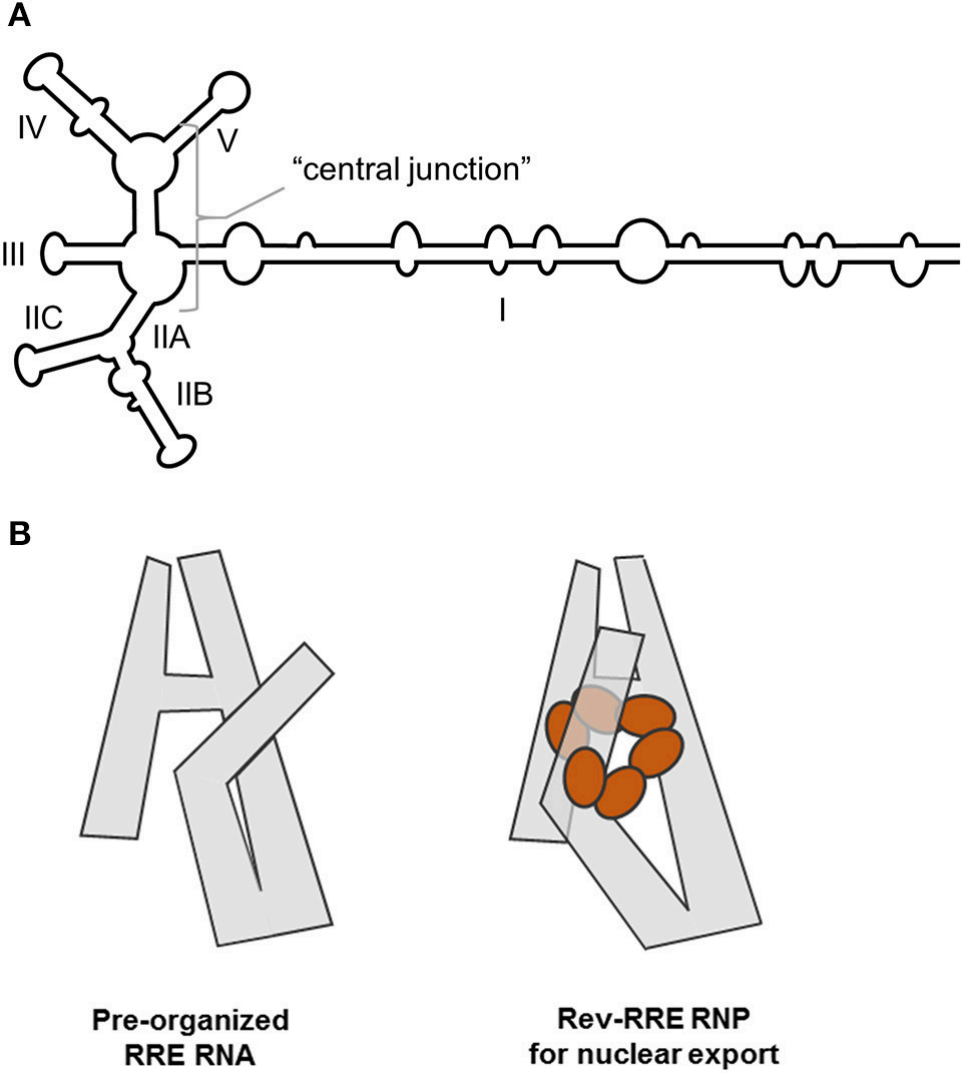
**(A)** Cartoon representation of a 5-SL form of the HIV-1 RRE with basepairing in the central junction. Stem, stem loop and central junction designations are indicated. **(B)** SAXS, SHAPE and aiSHAPE analysis suggests that the distal segment of stem I (S-I) folds back on itself to provide a cryptic Rev binding site, pre-organizing the RRE for optimal Rev multimerization. Three-dimensional RRE and Rev_6_-RRE complex model structures are shown. Adapted from Bai et al. (2014).

Enzymatic and chemical probing methods, including SHAPE, have been used to show that the HIV-1 RRE assumes a structure characterized by either 4- or 5- stems and stem loops arranged around a central junction (Figure 1A; Olsen et al., 1990; Kjems et al., 1991; Mann et al., 1994; Lusvarghi et al., 2013a; Pollom et al., 2013; Bai et al., 2014). Depending on the HIV strain of origin, the RRE central junction may itself be partially basepaired (Figure 1A) in a manner resembling the HIV-2 and SIV RREs (Lusvarghi et al., 2013a; Pollom et al., 2013; Bai et al., 2014). Stem I (S-I) is formed by hybridization of 5′ and 3′ terminal RRE sequences, and is by far the longest of the RRE sub-motifs in every published secondary structural model. SL-II bifurcates into SL-IIA and SL-IIB, with the latter motif serving as the primary Rev binding site (Olsen et al., 1990; Heaphy et al., 1991; Malim and Cullen, 1991; Iwai et al., 1992; Tiley et al., 1992; Pond et al., 2009). A second high affinity binding site is located within an internal loop of S-I proximal to the central junction (Daugherty et al., 2008), and a “jellyfish” model of cooperative Rev assembly initiating from these sites has been proposed (Daugherty et al., 2010). According to this model, six rev molecules cluster around a common face of the HIV-1 RRE while their intrinsically unfolded C-terminal effector domains are arranged in parallel on one side of the complex. A more recent structural study suggests that Rev binding at SL-IIB directs S-1 to fold back onto the central junction to form a cryptic, higher order Rev binding site and accelerate assembly of the Rev_6_-RRE complex (Figure 1B; Bai et al., 2014).

Establishing whether the RRE from NL4-3 HIV assumes a 4- or 5-stem configuration proved difficult until it was determined that the two forms are present in approximately equal proportions under defined solution conditions (Kjems et al., 1991; Sherpa et al., 2015). Independent resolution of the two structures by SHAPE was achieved by separating the conformers by non-denaturing polyacrylamide gel electrophoresis (ND-PAGE) and probing them *in situ*. The results of this analysis, together with a more detailed description of the method used to obtain them, will be discussed in greater detail in a subsequent section.

### *musD* MTE

**Figure 2:**
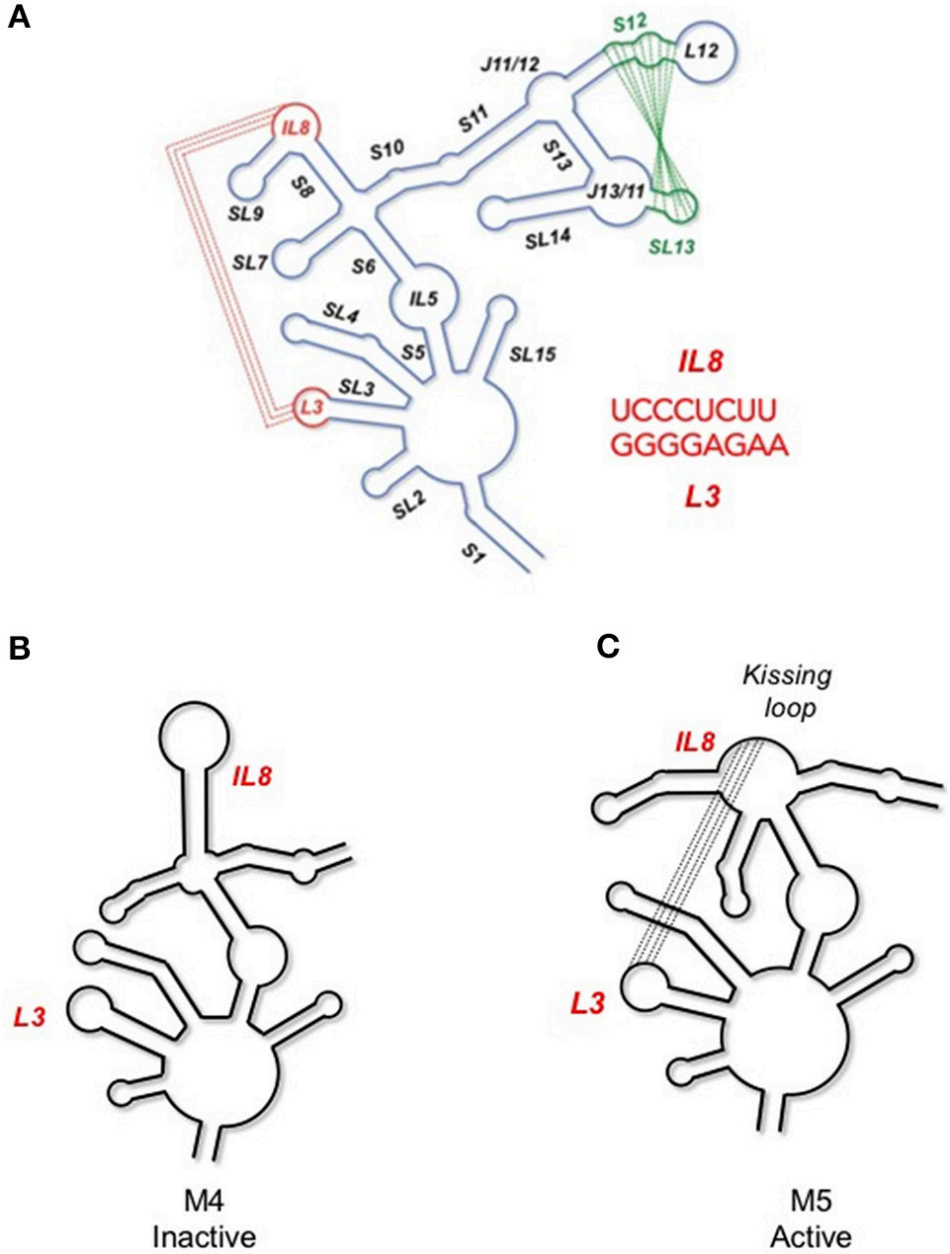
**(A)** Secondary structure of the musD, MTE, indicating long range interactions important for nucleocytoplasmic RNA transport. An annotated secondary structure is provided, where S, L, SL, and IL refer to stem, loop, stem-loop and internal loop, respectively. The L3/IL8 kissing interaction is indicated in red and the S13/SL12 pseudoknot in green. **(B)** Site-directed mutagenesis that interrupts the former interaction results in loss of MTE function (mutant M4). **(C)** In contrast, creating a substitute kissing loop interaction (mutant M5), restores activity, suggesting the importance of the kissing interaction in supporting overall MTE topology. See Legiewicz et al. (2010) for additional details on MTE function and mutagenesis.

Analogous to retroviruses and retroelements, the murine long terminal repeat retrotransposon, *musD*, also contains an RNA element (designated the *musD* transport element, or MTE) that likewise makes use of cellular export machinery (Smulevitch et al., 2005). In a comparative study, chemoenzymatic footprinting of the wild type *musD* MTE suggested a complex structure that contained both a kissing interaction and a pseudoknot (Legiewicz et al., 2010, Figure 2A). It was proposed that the former motif might serve as a platform for binding of specific cellular factors related to nuclear transport, or that it organizes the overall MTE structure to facilitate binding of cellular factors at other locations. MTE mutants M4 and M5 were constructed to address these possibilities (Figures 2B,C). In both cases, the original kissing interaction was disrupted. However, in mutant M5, MTE sequences were manipulated to introduce a novel kissing interaction in the immediate vicinity. Using a model reporter assay, mutant M4 was unable to support nucleocytoplasmic RNA transport, whereas mutant M5 retained full biological activity. Taken together, these data suggest that similar to the stem I of the HIV RRE, the kissing complex might play a role in pre-organizing the MTE into a configuration that supports binding of host factors.

### MLV PTE

**Figure 3:**
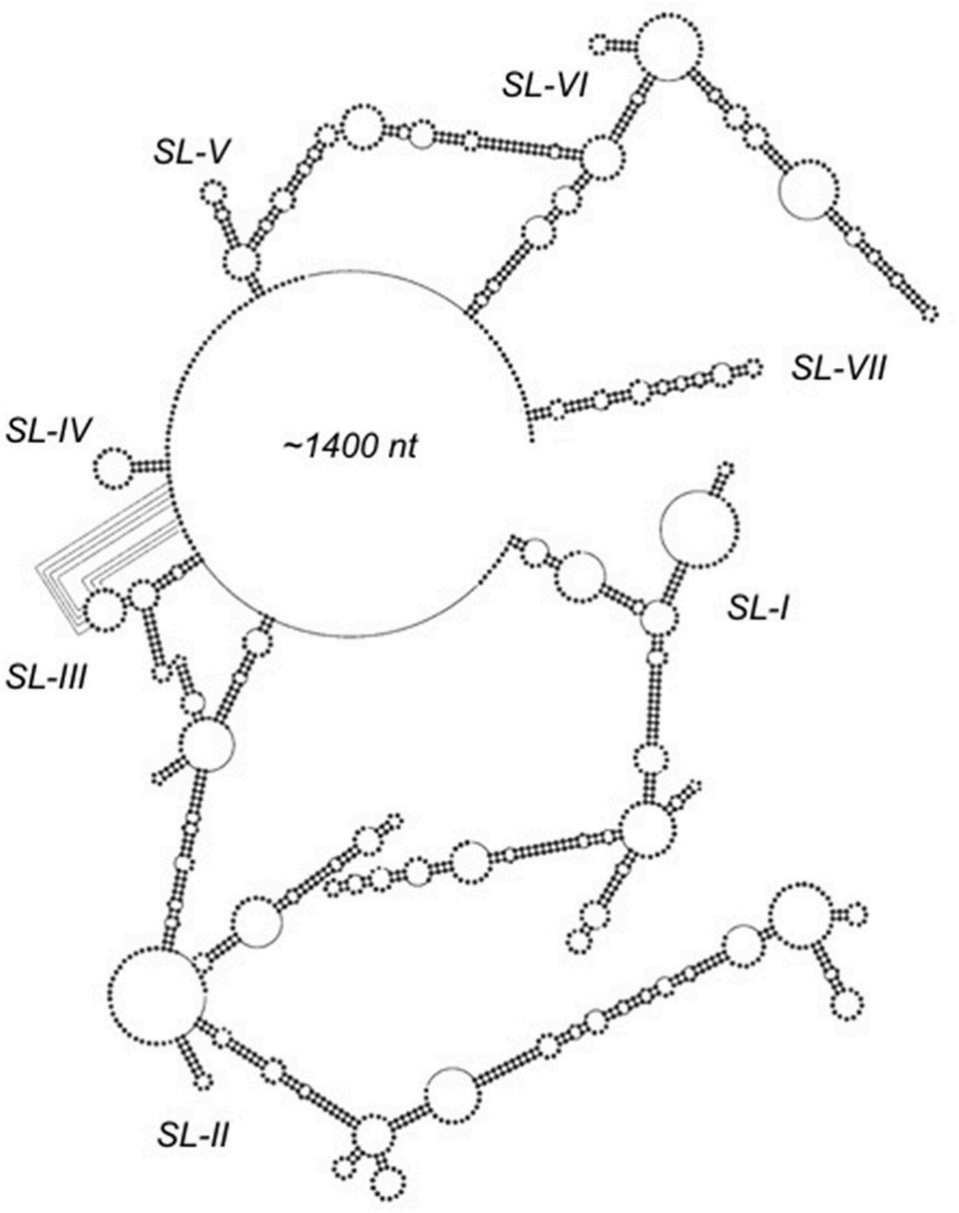
SHAPE-predicted secondary structure analysis of the ~1,400 nt gammaretroviral RNA transport element, PTE. Stem-loops (SL) SL-I through SL-VII are indicated. Proposed long range interactions involving nucleotides of SL-III are indicated by dotted lines. Adapted from Pilkington et al. (2014).

In contrast to complex retroviruses, simple retroviruses produce only two mRNAs encoding the *gag/pol* and *env* genes, respectively. As examples of nucleocytoplasmic RNA transport elements in the gammaretrovirus family, we determined the structure of the post-transcriptional element (PTE) of murine leukemia virus (MLV) and xenotropic murine leukemia virus-related virus (XMRV), which share 98% homology and can functionally substitute for HIV-1 Rev/RRE (Pilkington et al., 2014). However, in contrast to the HIV-1 RRE and *musD* MTE, the “simple” gammaretroviral PTE comprises a seven stem-loop structure spanning ~1,400 nt (Figure 3). Understanding the contribution of each stem-loop to nuclear export required a series of deletion experiments. Removing the apical half of SL II did not appreciably affect nuclear export, while deleting it entirety reduced activity by 50%, indicating that SL-II is not essential for PTE function. In contrast, deletion of SL-I or SL-VII resulted in complete loss of export activity. The presence of SL-I and SL-VII alone, however was insufficient to support nuclear export, demonstrated by experiments with minimal SL-I/SL-VII constructs. Intervening motifs, therefore, likely play a role in positioning SL-I and SL-VII for recognition by cellular export factors.

## Tackling challenges of conformational heterogeneity

Interpretation of SHAPE-derived structural data assumes that RNAs under investigation are structurally homogeneous; unfortunately, this is not always the case. Whenever possible, SHAPE experiments should be preceded by an evaluation of structural uniformity by non-denaturing polyacrylamide gel electrophoresis (ND-PAGE), wherein different conformers typically migrate at different rates. When multiple conformers are detected, site-directed mutagenesis can be used to try to force an RNA to assume a single structure; however, this approach frequently produces unexpected conformational changes that can further confound interpretation of SHAPE data. Alternative means of resolving the different structures assumed by an RNA have been developed, including (i) fractionating the conformers by ND-PAGE, excising the respective gel slices and then probing them separately *in situ*, or (ii) using ND-PAGE to quantify conformers present under differing experimental conditions and then applying this quantification to mathematically deconvolute reactivity values obtained under these conditions into conformer-specific profiles. The former approach, also referred to as in-gel SHAPE, was used to distinguish among alternative conformations assumed by monomeric and dimeric HIV 5′UTR constructs as well as the HIV-1 RRE. The latter method permitted characterization of the HIV-2 RRE as it assumed progressively more stable structural intermediates over time.

### HIV-1 genome dimerization

**Figure 4:**
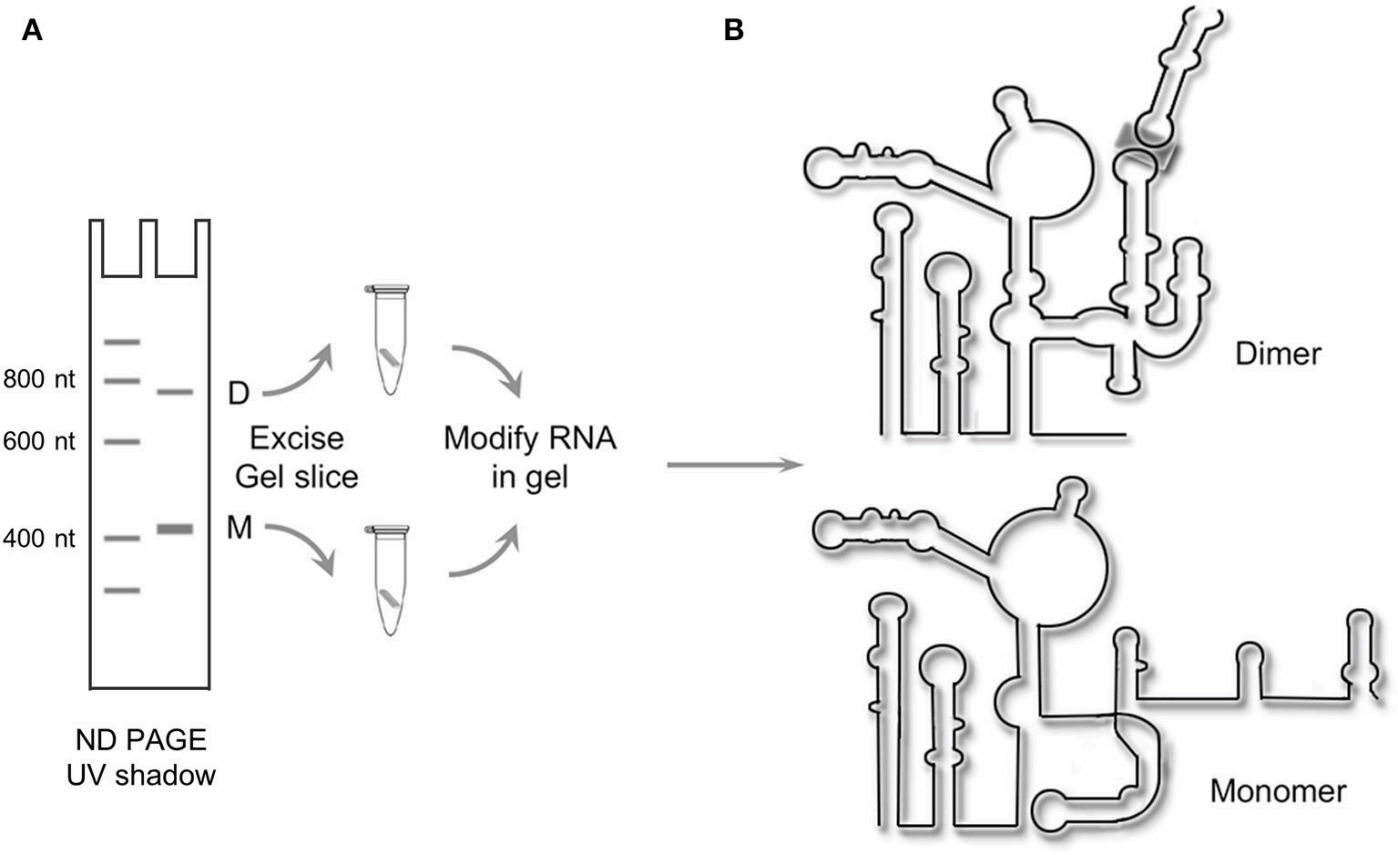
Resolving alternate conformers of the HIV-1 5′UTR by in-gel SHAPE. **(A)** Schematic depicting native polyacrylamide gel electrophoresis of *in vitro*-transcribed HIV-1 5′ UTR RNA to resolve the monomer (M) and dimer species (D), which are excised and treated with the SHAPE reagent *in situ*. **(B)** Following recovery of chemically modified RNAs, the SHAPE protocol is completed, revealing the monomeric and dimeric configurations of the 5'UTR construct. Adapted from Kenyon et al. (2013).

Dimerization of the (+) strand RNA genome is central to HIV packaging (Johnson and Telesnitsky, 2010) and is also critical for proteolytic processing of the Gag polyprotein during virion maturation (L'Hernault et al., 2012). Signals for HIV dimerization and packaging have been mapped to the 5′UTR, a construct of which was generated by *in vitro* transcription for structural analysis using CE-SHAPE (Kenyon et al., 2013). Such experiments were complicated, however, by the observation that the 5′UTR construct existed in an equilibrium state between monomer and dimer in solution (Figure 4A).

Following ND-PAGE, the two RNA species were visualized by ultraviolet illumination, excised and subjected to in-gel chemical modification with the slow-acting acylation reagent NMIA (Kenyon et al., 2013). Thereafter, modified RNAs were recovered by electro-elution and reverse transcribed, and the derivative cDNA products analyzed by capillary electrophoresis. The results of these experiments, as depicted in Figure 4B, indicate that the monomeric and dimeric forms of the HIV-1 5′UTR construct assume significantly different conformations. From this, a structural switch mechanism was proposed in which the palindromic dimer initiation sequence alternatively hybridizes to an intramolecular sequence in the monomeric RNA or its counterpart in a different monomer to form the dimer complex.

### HIV-1 RRE: resolution and significance of 4-SL and 5-SL conformers

As initially noted in section HIV-1 RRE: Overall structure and the role of SL-1, computational modeling and RNA probing methods, either alone or in combination, have alternatively supported 4-SL (Charpentier et al., 1997) or 5-SL (Kjems et al., 1991) RRE variants originating from NL4-3 or similar laboratory strains of HIV-1. Our own studies analyzing RRE structure in an HIV mutant resistant to *trans-*dominant RevM10 therapy, supported a 4-SL structure for the wild type RRE, but suggested that resistance-conferring mutations stabilized the 5-SL conformer (Legiewicz et al., 2008). In biophysical analyses, the 4-SL model best fits the 3-dimensional “A-like” RRE configuration predicted by small angle X-ray scattering (Fang et al., 2013), while atomic force microscopy suggests that the RRE exists in a state of equilibrium between 4-SL and 5-SL forms (Pallesen et al., 2009).

**Figure 5:**
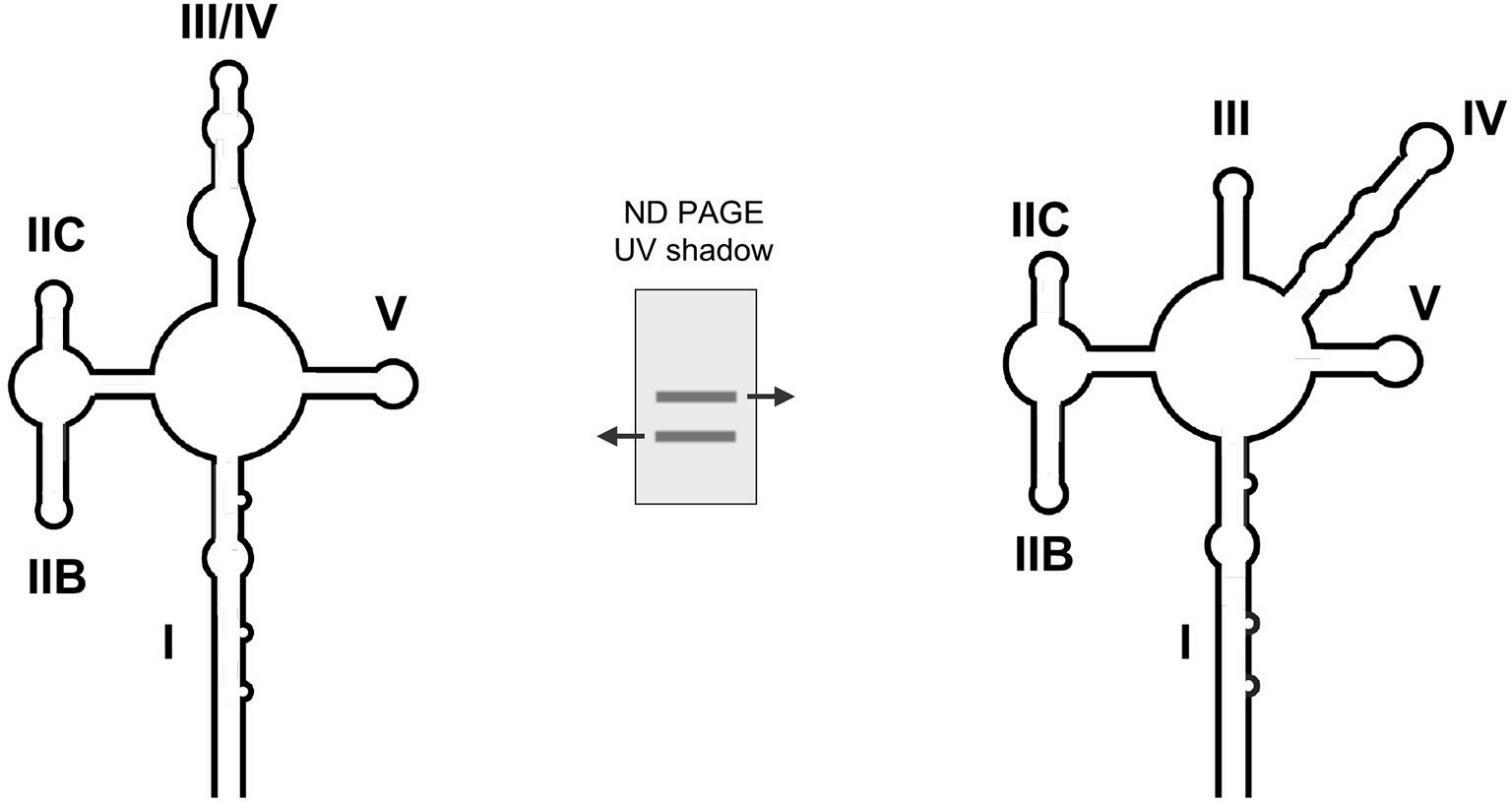
Dissecting alternative topologies for the HIV-1 RRE by native gel electrophoresis and in-gel SHAPE. The central panel illustrates that the RRE exists as an equilibrium mixture of two conformers that can be separated by prolonged electrophoresis. In-gel SHAPE indicates that the faster and slower migrating forms assume the 4-SL and 5-SL conformations, respectively. Adapted from Sherpa et al. (2015).

One finding that may reconcile these seemingly contradictory results comes from ND PAGE analysis of an HIV-1 RRE construct that revealed two closely-migrating species (Sherpa et al., 2015; Figure 5). Using in-gel SHAPE, it was determined that fast- and slow-migrating variants assumed the 4-SL and 5-SL conformations, respectively. To assess the biological significance of variable RRE conformation, mutant RREs that exclusively assumed the 4-SL or 5-SL structure were constructed, validated by CE-SHAPE, and evaluated in cell culture. Using a growth competition assay, where two or more viral variants compete for the same cell population under precisely the same environmental conditions (Quinones-Mateu et al., 2000), recombinant HIV-1 containing a “stabilized” 5-SL RRE was found to replicate more efficiently than both the “stabilized” 4-SL and wild type viruses. Although it remains to be determined experimentally, it has been suggested that alternate HIV-1 RRE conformers capable of sustaining different replication activities permit the virus to modulate its rate of replication under distinct conditions in order to better accommodate the host environment.

### Conformational transitions: the HIV-2 RRE

**Figure 6:**
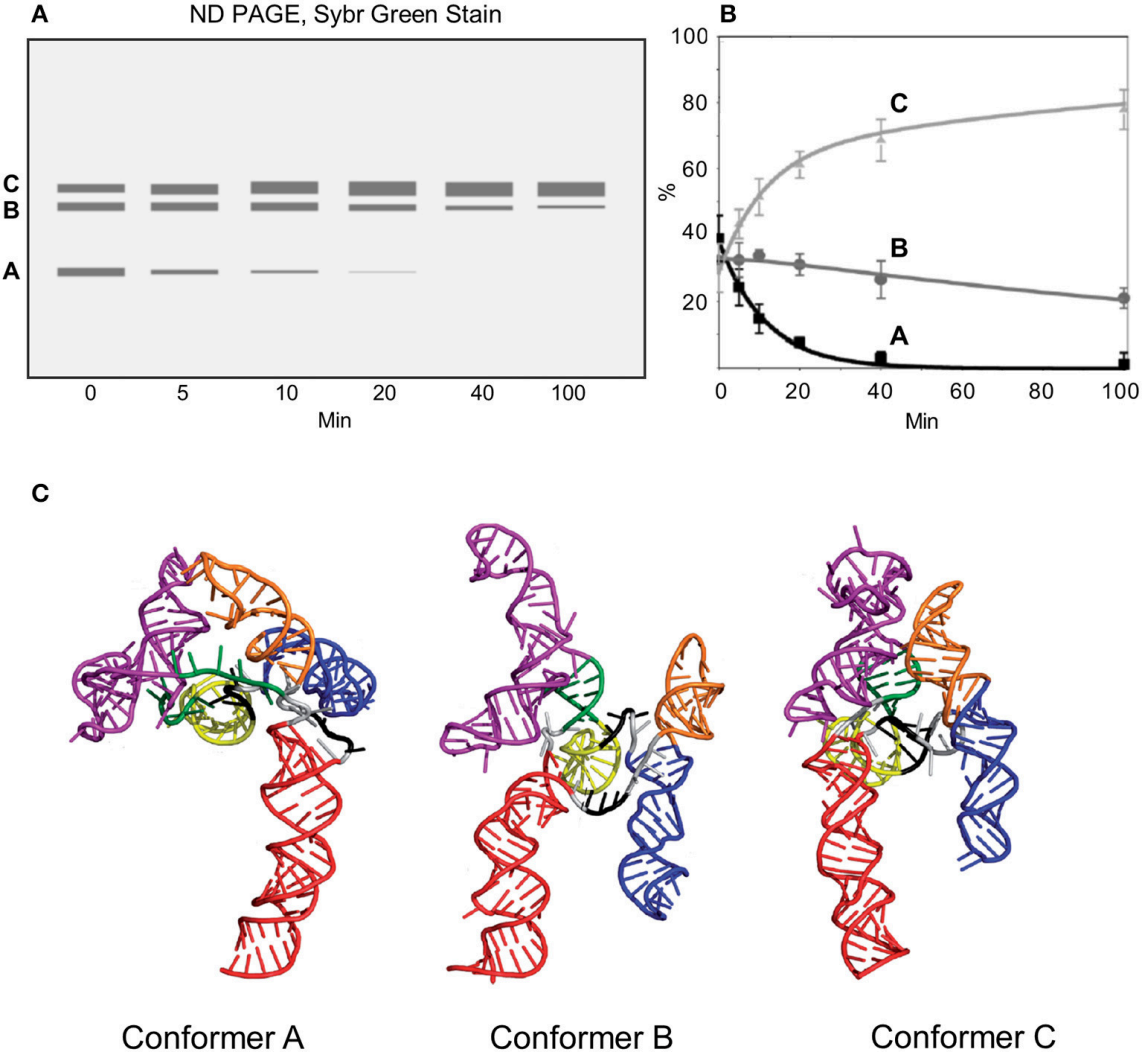
Analysis of HIV-2 RRE conformational changes during folding. *In vitro* transcribed RNA constructs are thermally denatured, flash cooled to 4°C and then incubated at 37°C. Immediately after flash cooling, the HIV-2 RRE exists as a mixture of A, B, and C conformations. Upon raising the temperature to 37°C, the relative ratios of the three variants changes over time, with conformer C ultimately predominating. **(A)** Schematic depicting non-denaturing gel electrophoresis of HIV-2 RRE as a function of incubation time. **(B)** Fluorometric quantification of fractionated RRE conformers. **(C)** Proposed models of the HIV-2 RRE open (Conformer A), intermediate (Conformer B) and closed forms (Conformer C). Secondary structural motifs are indicated and color-coded as follows: SL I, red; SL IIA, dark green, SL IIB, IIC and adjacent connecting loops, magenta; SL III, yellow; SL IV, blue; SL V, orange. Adapted from Lusvarghi et al. (2013a).

When *in vitro* transcribed RNA constructs containing the HIV-2 RRE are heated, flash cooled, and incubated at 37°C, the HIV-2 RRE proceeds through two conformational transitions before assuming the energetically most favorable form (Lusvarghi et al., 2013a, Figure 6A). These conformations can be segregated and quantified by ND-PAGE, although the RNA does not become structurally homogeneous until more than 100 min into the folding reaction. To structurally characterize the two transient intermediates as well as the final, most stable structure, a mathematical method was devised in which conformer-specific reactivity values were extracted from those obtained from mixtures of conformers by conventional CE-SHAPE. This strategy assumes that each ensemble reactivity value obtained from a mixture of RNA conformers equals the sum of values of the component conformers, weighted according to the fractional contribution of each to the total RNA population. Moreover, if ensemble NMIA reactivity values and fractional contributions of individual conformations change with folding time, and these values can be determined for a number of distinct times equal to or greater than the total number of conformers in the mixture, then specific reactivity values can be mathematically calculated for each conformer. In the case of the HIV-2 RRE, at 20 and 40 min after RNA folding was initiated, Conformers B and C constituted the predominant species, while the amount of conformer A was negligible. Thus, the following two-equation, two-variable system could be applied:

$$\begin{array}{l} R_{T20}=R_{B}p_{B20}\;{+\;R}_{C}p_{C20}\\ R_{T40}=R_{B}p_{B40}\;{+\;R}_{C}p_{C40} \end{array}$$

where *R* = the experimentally-determined ensemble NMIA reactivity for the nucleotide in question, *T* = time (in min) and *p* = fractional conformer contribution as determined by ND-PAGE (Figure 6B). By solving this system for *R*_*B*_ and *R*_*C*_ at each nucleotide position, NMIA reactivity values of the Conformers B and C were straightforwardly obtained. Subsequently, the following formula was used to obtain the reactivity of the A conformer at each nucleotide position:

$$\begin{array}{l} R_{T5}=R_{A}p_{A5}+R_{B}p_{B5}\;{+\;R}_{C}p_{C5} \end{array}$$

Ensemble reactivity value at 5 min (*R*_*T*5_), the % contribution of each conformer at 5 min (*p*_*A*5_*, p*_*B*5_*, p*_*C*5_), and the reactivity values of B and C (*R*_*B*_ and *R*_*C*_) were substituted into this equation to calculate the reactivity of A at each nucleotide position (*R*_*A*5_). Values obtained at 10 min (*R*_*T*10_, *p*_*A*10_*, p*_*B*10_, and *p*_*C*10_), together with the calculated values of *R*_*B*_ and *R*_*C*_, were substituted into an equivalent equation to obtain a second set of reactivity values for A (*R*_*A*10_). Finally, *R*_*A*5_ and *R*_*A*10_ NMIA reactivity values were averaged to obtain *R*_*A*_ values for determining the Conformer A structure. Combining extrapolated Conformer A, B, and C NMIA reactivity profiles with the folding algorithm RNAComposer (Popenda et al., 2012) produced 3D structures for “open,” “intermediate,” and “closed” variants of the HIV-2 RRE illustrated in Figure 6C.

## Characterization of long-range viral RNA interactions is facilitated by CE-SHAPE

In contrast to most chemical and enzymatic RNA probing methodologies, SHAPE permits probing of all four ribonucleotides and can be readily adapted to capillary electrophoresis. Consequently, base-pairing of nucleotides in relatively lengthy RNAs can frequently be determined in one or a few capillary electrophoresis-based SHAPE (CE-SHAPE) experiments—an advantage that significantly simplifies detection and characterization of long-range, *cis*-acting RNA interactions. Such interactions have been identified in positive-sense RNA plant viruses including members of Luteoviridae and Tombusviridae families, and human pathogens such as foot-and-mouth disease virus, hepatitis C virus (HCV), West Nile virus (WNV) and dengue virus (DENV) (Nicholson and White, 2014). Long-range interactions have also been identified in the negative-sense RNA genome of Ebola virus (EBOV), a filovirus that causes Ebola hemorrhagic fever in humans and other mammals (Sztuba-Solinska et al., 2013). This section will highlight two studies in which 5′-3′ interactions in DENV and EBOV were characterized using capillary electrophoresis-based SHAPE (CE-SHAPE) and complementary biochemical techniques (Sztuba-Solinska et al., 2013, 2016).

### Dengue virus

As is characteristic of flaviviruses, the DENV genome encodes a single-open reading frame flanked by highly structured untranslated regions (UTRs) (Villordo and Gamarnik, 2009; Wei et al., 2009; Filomatori et al., 2011). This positive-sense RNA also provides a template for translation of the viral polyprotein precursor or for RNA-dependent synthesis of negative-sense DENV RNA, which is then used to regenerate the genomic RNA strand. The secondary structures of the positive-sense DENV RNA UTRs are of particular interest, as these elements have been shown to contribute to regulation of translation, replication, transcription and viral pathogenesis (Pijlman et al., 2008; Wei et al., 2009; Manzano et al., 2011). RNA folding algorithms and phylogenetic comparisons have been used to identify putative functional motifs in DENV UTRs (Olsthoorn and Bol, 2001; Manzano et al., 2011), but these predictions were not experimentally validated. In addition to the advent of SHAPE technology, study of DENV replication and RNA structure mapping has been greatly facilitated by the availability of a 719-nt minigenomic DENV RNA construct (DENV-MINI) that houses both UTRs, is replication competent in cell culture and can be produced by *in vitro* transcription (You and Padmanabhan, 1999).

**Figure 7:**
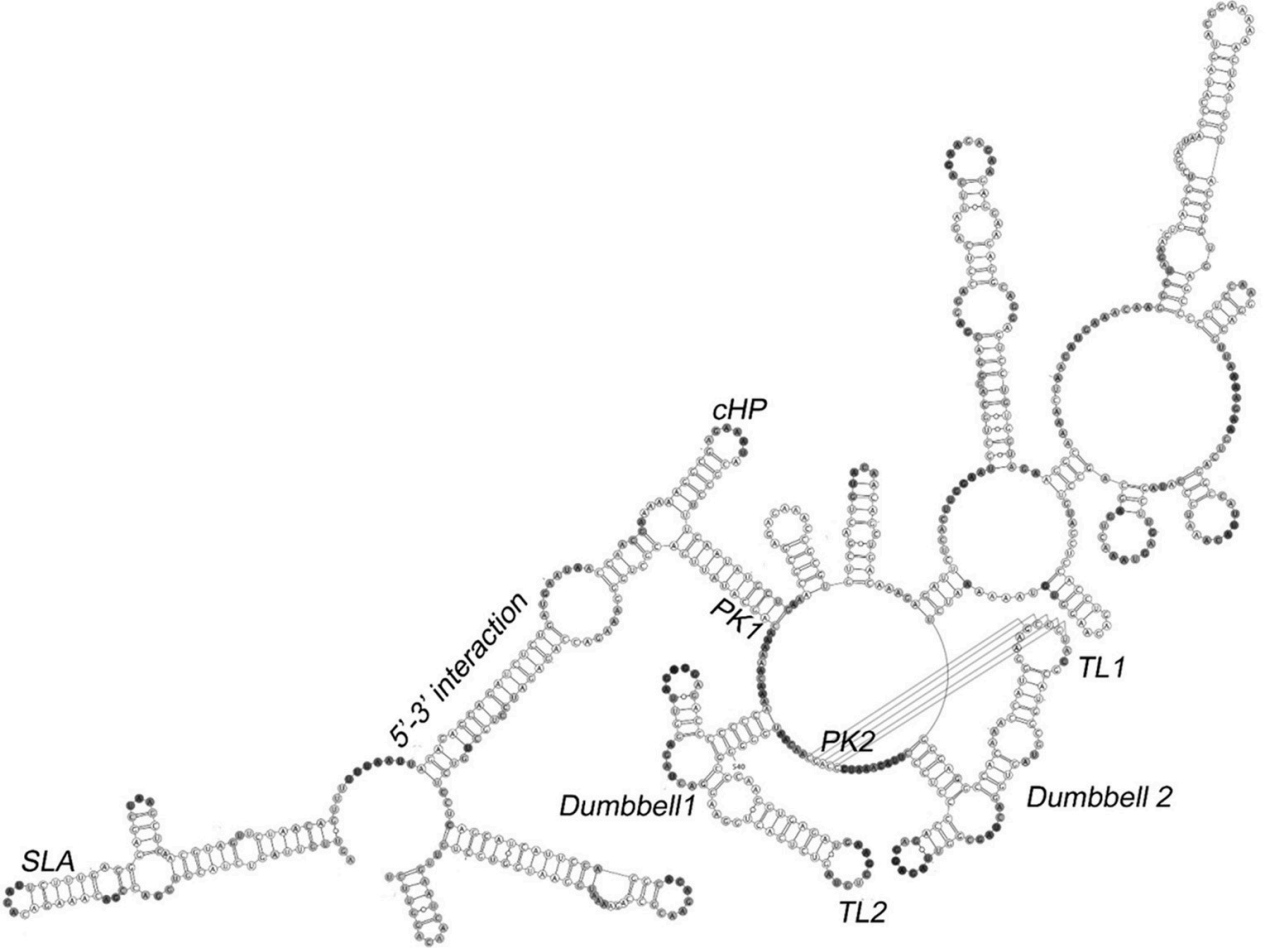
Secondary structure of the DENV minigenome determined by CE-SHAPE. Stem loop A (SLA), dumbbell, pseudoknot (PK1 and PK2), terminal loop (TL1), capsid-coding region hairpin element (cHP) and 5′-3′ interaction motifs are indicated. Despite the designation, neither CE-SHAPE nor aiSHAPE indicated formation of a pseudoknot involving the PK1 motif. Nucleotide shading is in proportion to normalized reactivity. Adapted from Sztuba-Solinska et al. (2013).

A secondary structural model of *in vitro* transcribed DENV-MINI RNA obtained by CE-SHAPE is presented in Figure 7 (Sztuba-Solinska et al., 2013). The construct can be divided into regions characterized by interactions between the 5′ and 3′ UTRs (the 5′-3′ UTRs region), the presence of two dumbbell and/or pseudoknot motifs (the Dumbbells region) and incomplete or artifactual interactions among segments of the truncated polyprotein coding segment of the DENV-MINI construct (the Variable region). Stem-loop A (SLA) and the 3′ stem loop (3′ SL) within the 5′-3′ UTRs region are located at or near the 5′ and 3′ termini of DENV-MINI, respectively. In line with mutational and molecular biological studies, the former motif has been implicated in positioning the DENV RNA-dependent RNA polymerase for initiation of negative sense RNA synthesis at the 3′ terminus (Lodeiro et al., 2009; Polacek et al., 2009). Base pairing between the 5′ and 3′ UTRs occurs within the adjacent dsUAR (double-stranded upstream of AUG region) and 5′-3′ cyclization sequence (CS) motifs, and the intervening capsid coding region hairpin element (cHP) reportedly essential for viral RNA synthesis (Clyde et al., 2008; Polacek et al., 2009).

The Dumbbells region is comprised of tandem dumbbell-shaped motifs and adjacent sequences (PK1, PK2) complementary to the respective apical loops. Mutational analysis of these motifs indicates that they are essential for both RNA replication and optimal translation (Manzano et al., 2011). Using aiSHAPE, a targeted antisense hybridization technique that will be discussed in greater detail in the next section, it was determined that basepairing between PK2 and the apical loop of the 5′-dumbbell (5′ DB) contributed to a pseudoknot, whereas the PK1 sequence (nt 614–624) paired with a remote segment of RNA (nt 134–144) to form the 5′-3′ CS motif. This observation was validated by mutational analysis and 3D modeling, the latter suggesting that spatial constraints in the Dumbbell region would render formation of the putative PK1 and PK2 pseudoknots mutually exclusive. The 3′ dumbbell and PK2 pseudoknot appear to support the overall structure of the DENV minigenome while perhaps also serving to position the 5′ and 3′ UTRs in proximity to facilitate negative-sense RNA initiation. Moreover, it has been postulated that the PK1 pseudoknot, while not observed in the *in vitro* transcribed DENV-MINI RNA, may form at other points in the DENV life cycle. For example, the motif may assemble either shortly following initiation of negative-sense RNA synthesis as secondary structural elements are sequentially disrupted, or during subsequent propagation and displacement of positive-sense genomic RNA from the (–) RNA template.

### Ebola virus

In contrast to Dengue and other positive-sense RNA viruses, the EBOV genome is an ~19 kB negative-sense RNA housing 7 anti-genes that are individually transcribed into positive-sense mRNAs for translation into viral proteins. This collective coding domain is flanked by non-coding 3′ leader and 5′ trailer regions that contain signals for anti-genome and genome replication. For convenience and safety, studies of EBOV replication are often conducted using reconstituted replication and transcription systems wherein transcription of a truncated genome containing a reporter gene flanked by leader and trailer sequences is complemented by expression of viral proteins *in trans*. One such model system was used in conjunction with CE-SHAPE and other probing techniques to map the secondary structure of the EBOV UTRs, including putative leader-trailer base-pairing interactions (Mühlberger et al., 1996, 1999; Watanabe et al., 2004; Uebelhoer et al., 2014).

**Figure 8:**
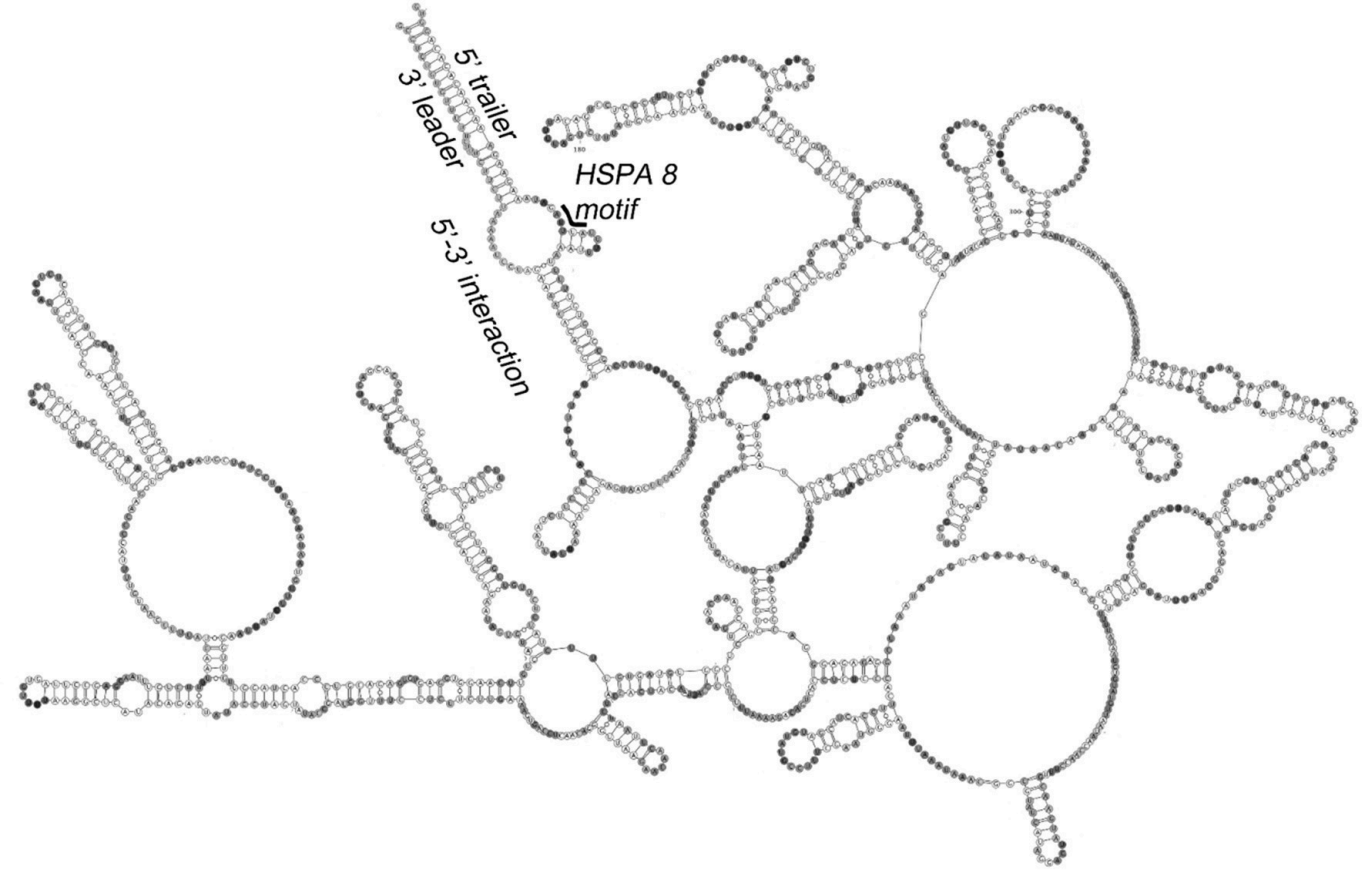
Secondary structure of the EBOV minigenome determined by CE-SHAPE. Predicted base pairing of 3′ leader and 5′ trailer sequences is shown together with a heat shock protein A 8 (HSPA 8) binding site. The discontinuity created by omission of the GFP gene from the displayed minigenome construct sequence is also indicated. Nucleotide shading is in proportion to normalized reactivity. Adapted from Sztuba-Solinska et al. (2016).

The secondary structure of a minigenomic EBOV RNA is depicted in Figure 8. This model is highlighted by the trailer-to-leader panhandle, a region of extensive basepairing between 5′ and 3′-terminal RNA, the existence of which was confirmed by mutational analysis and aiSHAPE (see below). The model is consistent with those of positive-sense RNA virus genomes like DENV, where bringing the RNA termini into proximity facilitates initiation of synthesis of the complementary strand. An alternative model of EBOV RNA structure, however, indicate that the 3′ leader does not interact with the 5′ trailer but instead houses a relatively short tandem hairpin motif to facilitate transcription initiation and genome replication (Schlereth et al., 2016). Although the competing structural models of the EBOV UTRs appear to be incompatible, they may be reconciled by a more fluid version, where, for example, a trailer-to-leader panhandle is favored for RNA packaging into nascent virions, but where local hairpins become manifest in the 3′ leader prior to transcription and/or genome replication. Such structural fluidity is perhaps supported by observations of meaningful conformational variants and transitions in other viral RNA elements such as the HIV-1 and HIV-2 RREs (Lusvarghi et al., 2013a; Sherpa et al., 2015).

## Using aiSHAPE to characterize RNA pseudoknots and kissing loops

One shortcoming of SHAPE and other biochemical probing methodologies is that, although the probabilities that individual ribonucleotides are basepaired can be calculated, their specific base pairing partners cannot. Typically, RNA secondary structural models are generated from SHAPE data using software such as RNAstructure, which converts reactivity values into pseudo-energy constraints that are superimposed onto the thermodynamic parameters used in the program's folding algorithm (Reuter and Mathews, 2010). The models thus produced represent RNA conformations calculated in aggregate as having the lowest pseudo-free energies. While this method has been demonstrated to dramatically increase the overall accuracy of RNA structure prediction, local structural motifs can easily be misrepresented in the model and cannot be independently verified without additional experiments. This is especially true when SHAPE is applied to large RNAs. Moreover, because early versions of the software were incapable of recognizing RNA tertiary elements such as pseudoknots and kissing loops, their existence could not be objectively determined.

In the absence of more direct methods, important RNA motifs are often verified using a mutational approach; i.e., the composition of the putative motif is altered by substitution or deletion of proximal nucleotides, and the consequent effects on RNA secondary structure are observed. While this approach can be useful, an important limitation is that even small alterations in RNA sequence can produce marked structural changes throughout an RNA, making it difficult to draw definitive conclusions regarding the role(s) played by the mutated nucleotides in maintaining the structure of the motif in question. AiSHAPE was developed as a complement to the site-directed mutagenesis, where the sequence of the RNA of interest is not altered (Legiewicz et al., 2010). Briefly, aiSHAPE involves incubating RNA with locked nucleic acid (LNA)-DNA chimeras usually designed to hybridize to a segment of the motif believed to be base-paired. These chimeras are typically 6–9 nt long, are interspersed with 3–4 LNA monomers, and bind with sufficient affinity to the target RNA that they displace nucleotides natively hybridized to the target and cannot themselves be displaced by the translocating polymerase during reverse transcription. Consequently, the secondary structures of individual motifs can be validated when previously unreactive basepairing partners of the LNA-DNA target sequence are displaced by the chimera and the reactivity of displaced nucleotides increases.

**Figure 9:**
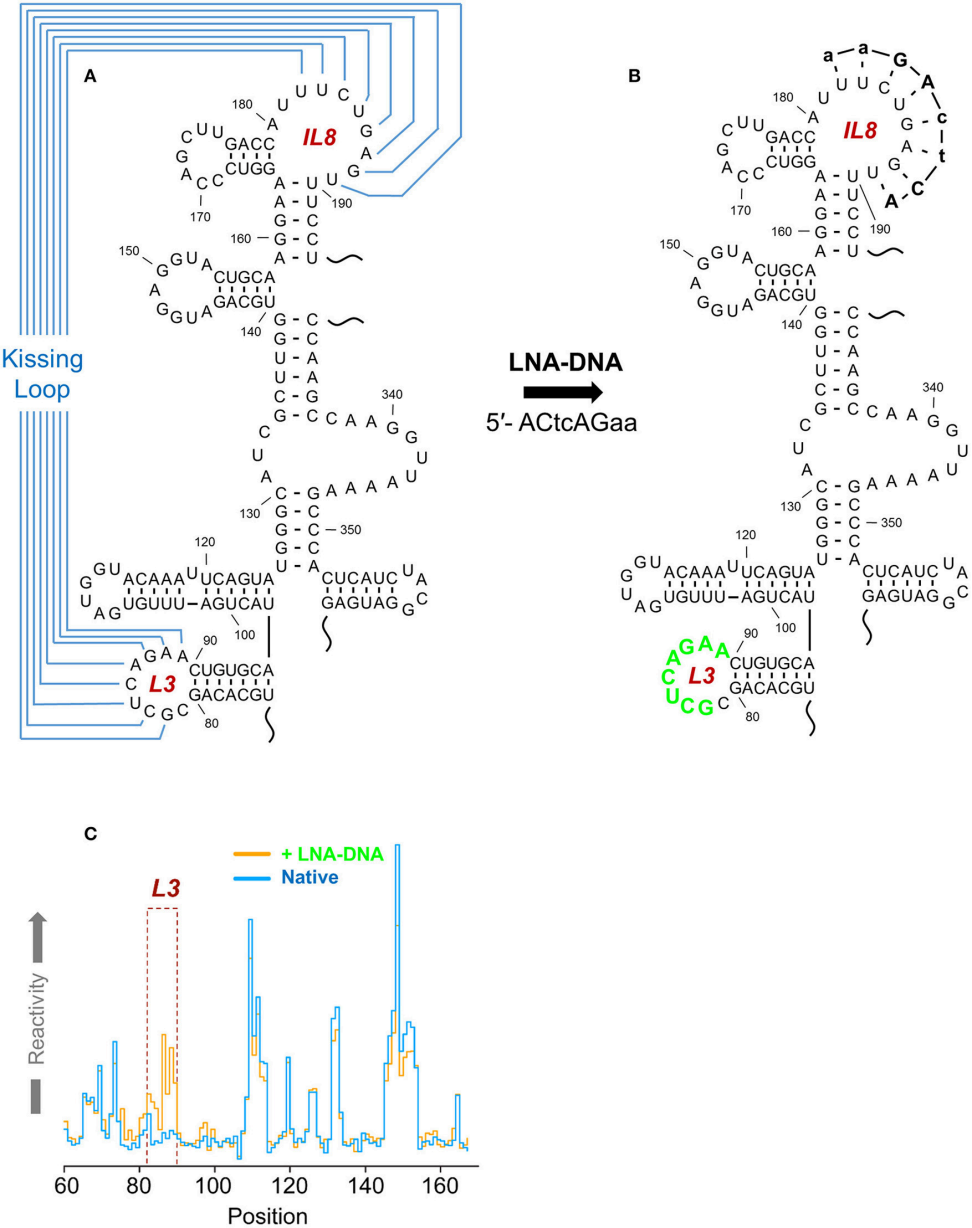
A kissing loop motif within the musD RNA transport element validated by aiSHAPE. **(A)** Predicted secondary structure the musD RNA transport element, including a postulated kissing loop interaction between nt 82–89 and nt 182–189. **(B)** The LNA-DNA chimera designed to hybridize to nt 182–189 would be expected to displace nt 82–89 and render them more susceptible to acylation. Perturbation of RNA structure outside of this region should be minimal. **(C)** A step plot comparing RNA reactivity values obtained in the presence and absence of the LNA-DNA chimera. A marked increase in reactivity was observed for nt 82–89 in the presence of the chimera, thus validating the kissing loop prediction. Adapted from Legiewicz et al. (2010).

AiSHAPE was first used to validate both a kissing loop and a pseudoknot in the minimal endogenous type D murine LTR retrotransposon (*musD*) RNA transport element (Figures 2, 9; Legiewicz et al., 2010). Mutational analysis proved that these tertiary interactions were essential for nuclear export of retrotransposon RNA. In the Ty1 retrotransposon, a pseudoknot was identified near both a palindromic RNA dimerization interface and the primer binding site (PBS), where the cognate tRNA-Met_i_ hybridizes to prime (–) DNA synthesis. In a separate study, this element was mutationally verified and demonstrated essential for transposition (Bolton et al., 2005; Purzycka et al., 2013). LNA-DNA chimeras were used in a related manner to block RNA dimerization and thus facilitate secondary structural mapping of the HIV 5′ UTR both by SHAPE and in three dimensions (Stephenson et al., 2013). Finally, aiSHAPE was used to validate or refute the existence of putative pseudoknot or hairpin motifs in the structural characterizations of DENV (Figure 7) and EBOV RNAs (Figure 8) described above.

## SHAPE-MaP and RNA structure probing *in Vivo*

Probing RNA in its native context, i.e., within cells or virions, requires assay sensitivity and throughput difficult to achieve with CE-SHAPE. For example, 20 L of cell culture and ~25 separate CE-SHAPE experiments were required to fully resolve the secondary structure of the HIV-1 genome (Watts et al., 2009). Although a remarkable accomplishment, such resource-intensive experimentation is beyond the means of most laboratories. Moreover, this work was possible only due to the high levels of target RNA enrichment provided in harvested virions. Due to the limited sensitivity and throughput of CE-SHAPE, a parallel study of any but the most abundant of cellular RNAs would be challenging.

An elegant solution to these shortcomings was provided in the form of SHAPE and mutational profiling, or SHAPE-MaP (Siegfried et al., 2014). In this adaptation of SHAPE, reverse transcription conditions are modified so that an acylated ribonucleotide causes misincorporation rather than premature termination of cDNA synthesis. Consequently, the resulting cDNA library contains mutations at sites opposite modified ribonucleotides and in proportion to the degree of acylation of those nucleotides in the RNA population. Also, because reverse transcriptase reads through the acylation sites, cDNA products can be amplified by PCR, which is subsequently used to add the required next generation sequencing (NGS) adapters. The resulting amplicon libraries are subjected to NGS and the numbers and locations of mutations are counted and automatically processed into SHAPE reactivity values *via* ShapeMapper software (Siegfried et al., 2014).

Amplification of sparse cDNA libraries by PCR renders SHAPE-MaP several orders of magnitude more sensitive than conventional CE-SHAPE, thereby bringing *in vivo* RNA structure mapping back into the realm of possibility. For instance, in the first application of SHAPE-MaP, the technique was used to repeat the structural analysis of HIV-1 genomic RNA from ten-fold fewer harvested virions than were required for the original CE-SHAPE study. Moreover, the “random priming” and “amplicon” variations of the method permit analysis of long RNAs in only one or a few NGS sequencing runs. Using Superfold software in conjunction with ShapeMapper, Shannon entropy calculations (Huynen et al., 1997) were newly applied in this study to identify the most structurally homogeneous segments of viral RNA, while ShapeKnots software (Hajdin et al., 2013) was used in conjunction to identify numerous previously unrecognized pseudoknots in the HIV-1 RNA sequence.

In separate work, SHAPE-MaP has been used to identify protein binding sites on RNase MRP, SRP RNA, 5S rRNA (Smola et al., 2015), and Xist RNA (Smola et al., 2016) *via* a strategy resembling the subtractive approach applied in classical nucleic acid footprinting techniques. Specifically, RNA is incubated with acylating reagent both in its native context (e.g., cells or virions, where proteins are present) and *ex vivo*, where proteins have been removed by organic solvent extraction. SHAPE-MaP reactivity values obtained from the two RNA samples are subsequently compared, with reduced values in the presence of protein considered as regions of protection attributable to protein binding, and the inverse result indicating a local, protein-induced increase in RNA flexibility. Collectively, these techniques were used to characterize the overall secondary structure and structural homogeneity of KSHV polyadenylated nuclear (PAN) RNA, as well as to map the binding sites of select viral proteins (Sztuba-Solinska et al., 2017).

The double-stranded DNA genome of KSHV is ~165 kB in length, with an associated transcriptome comprised of ~90 protein-coding genes and numerous non-coding RNAs, including PAN. PAN RNA is ~1.1 kB in length, and although primarily localized to the nucleus (Sun et al., 1996) can also be isolated from KSHV virions and the cytoplasmic fraction of infected cell extracts (Bechtel et al., 2005; Rossetto et al., 2013). This long non-coding RNA (lncRNA) is produced during lytic KSHV infection and suppresses expression of host genes involved in the antiviral response, probably *via* interactions with transcriptional regulators, chromatin modifiers and other host and viral proteins (Rossetto and Pari, 2011, 2012). Numerous sites of PAN interactions with the viral episome have also been identified, suggesting an additional role for this lncRNA in regulating KSHV protein expression (Rossetto et al., 2013). PAN has evolved multiple strategies to avoid cellular decay, one of which utilizes the expression and nuclear retention element (ENE), a U-rich, 3′-terminal motif that sequesters the poly-A tail to form a triple helix as a means of conferring resistance to nucleolytic degradation (Conrad and Steitz, 2005; Conrad et al., 2006; Mitton-Fry et al., 2010; Borah et al., 2011; Massimelli et al., 2011; Sei and Conrad, 2011; Rossetto et al., 2013). In addition, binding of the KSHV ORF57 to the PAN mRNA transcript accumulation protein responsive element (MRE) both confers RNA stability and promotes nuclear retention of PAN RNA (Massimelli et al., 2011; Sei and Conrad, 2011).

**Figure 10:**
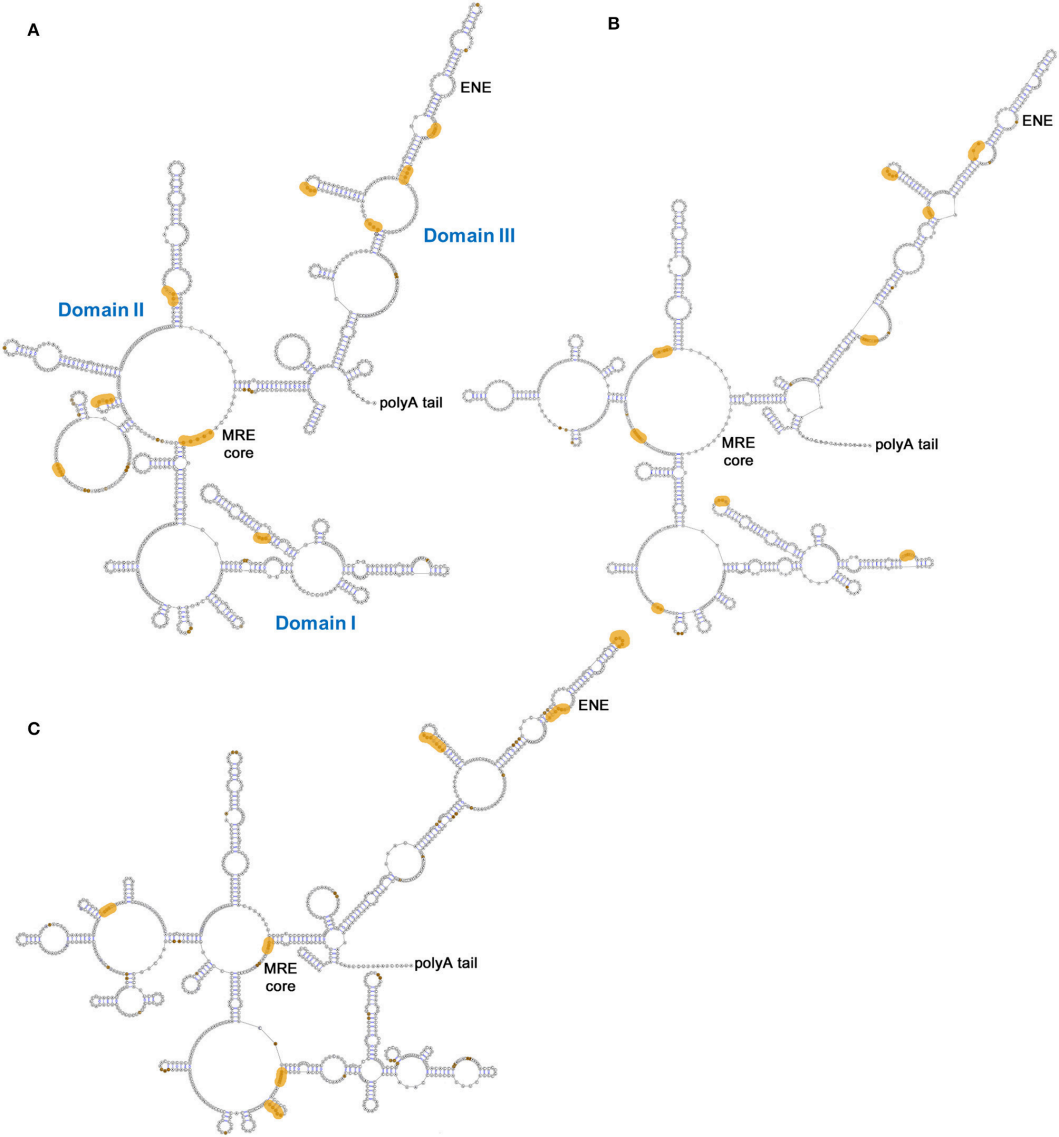
PAN RNA secondary structures and potential KSHV protein binding sites. Models generated from SHAPE-MaP experiments using PAN RNA isolated from **(A)** nuclei, **(B)** cytoplasm or **(C)** virions. The MRE core motif and poly-A tail are indicated in all panels, while Domains I-III are common to all three panels but are indicated only in panel **(A)**. Orange zones reflect differences in reactivity calculated for RNA probed *in*- or *ex-vivo* that are consistent with protein binding. Adapted from Sztuba-Solinska et al. (2017).

One of the principle achievements of the PAN RNA structural study was using SHAPE-MaP to characterize PAN isolated from infected cell nuclei, cytoplasm, and KSHV virions (Figure 10). Each of these structural variants is divided into three domains (I-III) separated by helical motifs. Of these, Domain III, which contains the ENE motif, is the most conserved across cellular and viral compartments—an observation supported by Shannon entropy calculations. Although the existence of the ENE triple helix could not be directly confirmed using SHAPE-MaP, the secondary structural models are all consistent with the duplex structure predicted for the conserved element. Binding sites for KSHV proteins on PAN RNA were also identified, both generally and with respect to individual viral proteins. Importantly, ORF57 binding mapped to the MRE and near the ENE in Domain III, in agreement with previous reports (Massimelli et al., 2011; Sei and Conrad, 2011). However, the MRE site was found to lack its binding partner in RNA extracted from the cytoplasm, indicating that ORF57 binding is indeed associated with nuclear retention. Other KSHV proteins (ORF73/LANA, ORF59, and ORF26) implicated in direct or indirect binding to PAN (Bechtel et al., 2005; Campbell et al., 2014; Rossetto and Pari, 2014) produced reactivity differences in numerous places upon binding to *in vitro* transcribed RNA, including at common sites at or near the MRE, within a large internal loop of Domain I and at the helical base of Domain III. Overlap among protein binding sites suggests that these motifs may individually or collectively seed formation of multi-protein RNA complexes in the context of the KSHV life cycle.

## Conclusion

SHAPE-MaP permits high-throughput secondary structure mapping of lengthy, sparse RNAs in their native contexts. In conjunction, ShapeKnots and Shannon entropy calculations allow researchers to identify otherwise hidden pseudoknots as well as structural motifs likely to be conserved during RNA conformational sampling. Acylating reagents used for SHAPE may also be used to map protein binding sites in the context of these experiments. For more routine analyses of short- and intermediate-length RNAs, and to avoid the costs associated with next generation sequencing, CE-SHAPE offers a practical alternative for identifying and characterizing RNA structural motifs that can be independently verified using the aiSHAPE antisense technology. Moreover, in-gel SHAPE and mathematical deconvolution of mixed reactivity profiles provide utility in examining heterogeneous and transitional RNA structures. Each of these approaches will continue to have value in the face of increasing demand for structural characterization of viral RNAs.

## Author contributions

JR, JS-S, and SL wrote and edited the manuscript and generated the figures.

### Conflict of interest statement

The authors declare that the research was conducted in the absence of any commercial or financial relationships that could be construed as a potential conflict of interest. The reviewer CR-L and handling Editor declared their shared affiliation.
